# Determinants of suicidal ideation in older adults: evidence from a national panel study in South Korea

**DOI:** 10.3389/fpubh.2025.1710375

**Published:** 2026-01-20

**Authors:** Eun-Young Park, Seung-Yi Choi, Jung-Hee Kim

**Affiliations:** 1 College of Education, Jeonju University, Jeonju, Republic of Korea; 2 College of Nursing, The Catholic University of Korea, Seoul, Republic of Korea

**Keywords:** depression, life satisfaction, older adults, self-esteem, suicidal ideation

## Abstract

**Introduction:**

This study aims to identify determinants of suicidal ideation (SI) among older adults in South Korea, with a specific focus on general characteristics, depression, self-esteem, and life satisfaction. Given the country's status as the fastest-aging OECD nation with the highest suicide rate among older adults, examining these factors is critical to informing public health strategies.

**Methods:**

Data were drawn from the 2023 wave of the Korea Welfare Panel Study (KoWePS), including responses from 5,736 individuals aged 65 years and more. Descriptive statistics and multivariate logistic regression analyses were conducted using SPSS 26.0 to identify significant predictors of suicidal ideation.

**Results:**

Findings revealed that age (OR = 2.442, *p* < 0.001), depression (OR = 0.920, *p* < 0.001), self-esteem (OR = 1.115, *p* < 0.001), and life satisfaction (OR = 1.095, *p* = 0.012) were significant predictors of suicidal ideation. In contrast, other general characteristics such as gender, income, and perceived health status were not statistically significant predictors. The study highlights the influence of sociocultural changes, including weakening of traditional family support systems, on mental wellbeing of older adults.

**Conclusions:**

Psychological and subjective wellbeing factors play a critical role in suicidal ideation among older adults. The study underscores the need for culturally sensitive screening and community-based interventions. Sociocultural shifts—such as weakening traditional family support systems—highlight the urgency of holistic, culturally informed suicide prevention strategies.

## 1 Introduction

Suicide represents a significant global public health concern, imposing not only emotional and societal burdens, but also substantial economic costs. In the United States alone, the socioeconomic cost of suicide and non-fatal self-harm is estimated to be approximately USD 510 billion annually (1). Globally, more than 720,000 individuals die by suicide each year (2). As the global population continues to age, suicide among the older adults are increasingly recognized as a major health issue (3–5). In particular, South Korea reports suicide rate among older adults of 46.6 per 100,000 population, significantly higher than the OECD average of 17.2 per 100,000 population, with the rate among those aged 80 and older being more than three times higher (6).

Suicide is not a singular act, but a complex and progressive process encompassing suicidal ideation (SI), planning, and attempts (7, 8). Numerous factors have been implicated in suicide among older adults, including mental health disorders, economic difficulties, somatic perception, and social isolation (2, 6). In terms of neurobiological factors, age-related alterations in serotonin neurotransmission, chronic neuroinflammation, and dysfunction of the prefrontal cortex—particularly in decision-making and emotional regulation—have been consistently associated with increased suicide risk in this population (9–13). Somatic complaints such as chronic pain, fatigue, and general physical discomfort have been found to correlate with elevated depressive symptoms in older adults. Studies conducted in Korean populations indicate that the presence and severity of physical symptoms are associated with an increased risk of both major depression and suicidal ideation (14, 15). These stressors often accumulate in later life, increasing psychological vulnerability and making older adults more susceptible to suicidal ideation (4, 16).

Empirical studies have shown that suicidal ideation in the older adults is closely associated with a combination of psychological and psychosocial variables such as depression, self-esteem, and life satisfaction (5). Depression has been consistently reported as the most prominent predictor, with increased severity of symptoms correlating with higher levels of suicidal ideation (17, 18). Depression in older adults often stems from experiences of loss, chronic illness, social withdrawal, and declining physical function. Self-esteem—a person's belief in their self-worth and ability to manage life's challenges—plays a protective role against suicidal ideation (19–21). Older adults with diminished self-esteem, particularly those facing social isolation and limited support, may perceive themselves as burdensome or incapable, which can increase their vulnerability to suicidal thoughts.

Life satisfaction is a core component of psychological wellbeing. It has been theoretically linked to reduced suicidal ideation, particularly among older adults. According to theories of subjective wellbeing and psychological resilience (22–24), individuals who maintain a high level of life satisfaction are better equipped to cope with age-related stressors such as physical decline, social isolation, and loss of autonomy—factors often associated with suicidal thoughts in later life. Empirical studies support this view, showing that life satisfaction can serve as a direct protective factor against suicidal ideation, independent of depressive symptoms. For example, Won et al. (25) have demonstrated that higher life satisfaction can significantly attenuate the association between depression and suicidal behavior in older adults, suggesting a buffering effect. Although research by Yu et al. (26) has focused on younger populations, their findings indicate that life satisfaction can negatively predict suicidal ideation, with depression acting as a partial mediator—implying a potentially similar psychological mechanism across age groups.

Notably, cultural characteristics unique to Korean society may further explain elevated levels of suicidal ideation among its older adults population. South Korea's traditional Confucian values emphasize filial piety, family cohesion, and adult children's responsibilities toward aging parents. However, these values have weakened due to industrialization and the rise of nuclear families (27). As a result, the proportion of Koreans who believe that families should be primarily responsible for supporting older adults has declined drastically—from 52.6% in 2007 to 21.39% in 2022 (28, 29). These cultural shifts might have contributed to older individuals feeling abandoned, burdensome, or purposeless, potentially increasing their risk for suicidal ideation.

Suicidal ideation, a phase in which individuals consciously contemplate ending their lives, is particularly critical because it serves as a gateway to more severe behaviors. It is generally understood as a continuum of thoughts about death and suicide, ranging from fleeting wishes to die to persistent thoughts of self-harm and concrete plans for suicide (3, 4, 8), and in the present study was conceptualized as the presence of such thoughts during the past year, regardless of their frequency or intensity. Therefore, identifying and addressing suicidal ideation among older adults is a crucial strategy in suicide prevention (30).

Although numerous studies have explored the relationship between suicidal ideation and individual psychological variables, most of them have focused on isolated factors without incorporating broader demographic and social dimensions (30, 31). Furthermore, research focusing specifically on the older adults population in Korea—who experience the highest suicide rate among older adults in the world (32)—remains relatively limited in scope and depth.

Therefore, the present study aimed to explore determinants of suicidal ideation among older adults in South Korea. Specifically, it examined effects of general characteristics, depression, self-esteem, and life satisfaction using a multivariate logistic regression approach based on nationally representative data.

## 2 Material and methods

### 2.1 Data

Based on the 2023 panel data from the Korea Welfare Panel Study (KoWePS), data relevant to this study's objectives were extracted. The Korean Welfare Panel started with 7,072 households in the first sample in 2006. The total number of households subject to the 18th survey was 8,008, including 1,800 new sample households in 2012 and 2,012 new sample households in 2022. Final panel households were selected using a double-sampling method. KoWePS panel data were collected through face-to-face interviews and self-report questionnaires. The total number of respondents through the 18th survey was 15,931, from which data of 6,161 people older than 65 years were extracted for analysis. After excluding those with missing values for depression, data of 5,736 people were analyzed.

Data accessed from the official homepage (https://www.koweps.re.kr) of KoWePS are freely available. The KoWePS is an ongoing longitudinal study of a nationally representative sample of South Korean households for which data are collected annually.

### 2.2 Measures

Age was divided into 65–80 years and older than 80 years. We defined the oldest-old as people aged ≥80 years following the definition by the United Nations (33). Age was entered into the logistic regression model as a binary categorical variable. Education level was divided into no education, graduate of elementary school, middle school, high school, college, university, and above graduate school. Income status was divided into low and general income. The equivalence scale was used for judging income status (34, 35). Low income indicates 60% below median value of the equivalized income, and general income indicates 60% above the median value. For analysis, income was dichotomized into these two categories. Married people were those with a spouse. Unmarried, bereaved, and divorced people were those without a spouse. Health status was measured on a 5-point Likert scale, ranging from very healthy to very unhealthy. Suicidal ideation (SI) was measured based on whether the individual had seriously thought about suicide in the past year, with response option of “yes” or “no.”

The CES-D scale used in the panel survey consists of 11 questions, each rated using a 4-point Likert scale. Among those questions, questions 2 and 7 were highlighted in the scoring process as measuring positive emotions, and these two questions were reverse scored according to the scoring guidelines. The higher the sum of scores for all questions, the more numerous their depressive symptoms. The reliability and validity of CES-D 11 have been confirmed by previous studies (36). Cronbach's α value of CES-D 11 in this study was 0.867.

Self-esteem was measured with Rosenberg's self-esteem scale (37) with the following ten items: (1) on the whole, I am satisfied with myself; (2) at times, I think I am no good at all; (3) I feel that I have a number of good qualities; (4) I am able to do things as well as most other people; (5) I feel I do not have much to be proud of; (6) I certainly feel useless at times; (7) I feel that I'm a person of worth, at least on an equal plane with others; (8) I wish I could have more respect for myself; (9) all in all, I am inclined to feel that I am a failure; (10) I take a positive attitude toward myself. The scale was answered on a four-point Likert scale ranging from strongly agree to strongly disagree. Five negatively worded items were reverse-coded. Cronbach's α value of Rosenberg's self-esteem scale in this study was 0.821 (range, 0.814–0.828).

Life satisfaction was measured using the following eight items: health satisfaction, family income satisfaction, residential environment satisfaction, family relationship satisfaction, job satisfaction, social relationship satisfaction, leisure life satisfaction, and overall satisfaction. It was assessed on a 5-point Likert scale ranging from strongly agree to strongly disagree. Its Cronbach's α value in this study was 0.810 (range, 0.803–0.817).

### 2.3 Statistical analysis

Descriptive statistics were used in this study to determine general characteristics of subjects. Multivariate logistic regression was conducted to analyze effects of general characteristics, depression, self-esteem, and life satisfaction on suicidal ideation among the older adults. Also, general characteristics of subjects such as gender, age, presence of spouse, income level, and health status was imputed in the multivariate logistic regression. SPSS 26.0 was used for all statistical analyses.

## 3 Results

### 3.1 General characteristics of participants

Table 1 shows general characteristics of the older adults. The sample included a higher proportion of females than males, and most participants were aged 65–80 years. Educational attainment was generally low, with elementary and middle school graduates comprising the largest groups. More than half of the participants belonged to the low-income category, and the proportion of married individuals was higher than that of unmarried individuals. Regarding health status, the majority reported average or poor health.

**Table 1:** General characteristics of participants (*n* = 5,737).

**Categories**	**Number of participants**		**Suicide thought**				
	***n***	**%**	**Yes**		**No**		***X** ^2^*
			***n***	**%**	***n***	**%**	
**Sex**							
Male	2,147	37.4	27	1.3	2,120	98.7	1.355
Female	3,590	62.6	59	1.6	3,531	98.4	
**Age**							
65–80	3,460	60.3	58	1.7	3,402	98.3	1.865
Above 80	2,277	39.7	28	1.2	2,248	98.8	
**Education level**							
No education	791	13.8	9	1.1	782	98.9	2.781
Graduate elementary school	2,368	41.3	41	1.7	2,327	98.3	
Graduate middle school	1,122	19.6	16	1.4	1,106	98.6	
Graduate high school	1,052	18.3	16	1.5	1,036	98.5	
Graduate college	76	1.3	1	1.3	75	98.7	
Graduate University	263	4.6	3	1.1	260	98.9	
Above Graduate school	61	1.1	0	0.0	61	100.0	
Missing data	4	0.1					
**Income status**							
General income	2,566	44.7	20	0.8	2,549	99.2	16.282^**^
Low income	3,171	55.3	66	2.1	3,105	97.9	
**Spouse**							
Yes	3,244	56.5	33	1.0	3,211	99.0	11.735^**^
No	2,493	43.5	53	2.1	2,440	97.9	
**Health status**							
Very health	66	1.2	0	0.0	66	100.0	68.709^**^
Healthy	1,578	27.5	7	0.4	1,571	99.6	
Average	1,988	34.7	19	1.0	1,969	99.0	
Not healthy	1,926	33.6	47	2.4	1,879	97.6	
Very not healthy	179	3.1	13	7.3	166	92.7	

^**^ *p* < 0.01.

Among general characteristics, factors that affected suicidal ideation were income level, presence of spouse, and health status. In terms of income level, the prevalence of suicidal ideation was higher in those with a low income than in those with a general income (*X*^2^ = 16.282, *p* < 0.01). The prevalence of suicidal ideation was higher in those without a spouse than in those with a spouse (*X*^2^ = 11.735, *p* < 0.001). In terms of health status, the prevalence of suicidal ideation was higher in those with poor health (*X*^2^ = 68.709, *p* < 0.001).

### 3.2 Factors affecting suicide ideation

Table 2 presents results of a logistic regression analysis of variables related to suicidal ideation, analyzing effects of gender, age, marital status, education level, income level, health status, depression, self-esteem, and life satisfaction on suicidal ideation. Gender did not significantly affect suicidal ideation (OR = 1.055, *p* = 0.845). However, the age had a significant effect, with the odds ratio of suicidal ideation increasing approximately 2.4 times higher in those aged above 80 compared to the reference group aged 65–80 (OR = 2.442, *p* < 0.001). Marital status (OR = 0.865, *p* = 0.577), education level (OR = 0.890, *p* = 0.242), and income level (OR = 0.692, *p* = 0.207) did not statistically significantly affect suicidal ideation. Health status showed a tendency to affect suicidal ideation. However, is effect being not statistically significant (OR = 0.754, *p* = 0.083). On the other hand, depression (OR = 0.920, *p* < 0.001), self-esteem (OR = 1.115, *p* < 0.001), and life satisfaction (OR = 1.095, *p* = 0.012) significantly affected suicidal ideation, showing that the more severe the depression, the higher the likelihood of suicidal ideation and the higher the self-esteem and life satisfaction, the lower the likelihood of suicidal ideation.

**Table 2:** Summary of logistic regression analysis for suicide thought (*n* = 5,737).

**Variable**	***B***	**SE**	**OR**	**−95% CI**	**+95% CI**	***p-*Value**
Gender	0.053	0.273	1.055	0.617	1.803	0.845
Age	0.893	0.255	2.442	1.481	4.025	< 0.001
Spouse	−0.145	0.260	0.865	0.520	1.439	0.577
Education level	−0.117	0.100	0.890	0.731	1.082	0.242
Income status	−0.369	0.292	0.692	0.390	1.227	0.207
Health Status	−0.282	0.163	0.754	0.548	1.038	0.083
Depression	−0.084	0.021	0.920	0.882	0.959	< 0.001
Self-esteem	0.109	0.032	1.115	1.047	1.188	< 0.001
Life satisfaction	0.091	0.036	1.095	1.020	1.176	0.012

B, unstandardized coefficient; SE, standard error; OR, odds ratio; CI, confidence interval.

## 4 Discussion

This study identified age, depression, self-esteem, and life satisfaction as significant predictors of suicidal ideation among older adults. While various general characteristics—including income level, marital status, and health status—were initially associated with suicidal ideation, multivariate logistic regression revealed that psychological and subjective wellbeing factors played a more critical role. Notably, older age was significantly associated with an increased likelihood of suicidal ideation, with each unit increase in age more than doubling the odds (OR = 2.442, *p* < 0.001), underscoring the heightened vulnerability of the older adults population. This trend aligns with previous research (3, 4, 30) and reflects compounding psychological, social, and existential pressures faced by aging individuals.

Korea's rapid transition into an aged society, coupled with inadequate adaptation of social and familial support structures, may help explain the country's disproportionately high suicide rate among older adults. According to the 2023 Older adults Status Survey, the majority of older adults reported a preference for a “well-dying” process that would minimize burden on others—highlighting how fear of dependency might intensify suicidal ideation in later life (38). While these cultural dynamics are particularly salient in Korea, similar patterns have emerged in other aging societies experiencing rapid social change. In countries such as Japan, China, and several Southern European nations, weakening of traditional family structures and reduction of intergenerational contact have been linked to increased psychological distress and suicidal ideation among older adults (39–41). The global shift from collectivist to more individualistic value systems often leaves older adults without expected family-based support, thereby increasing emotional isolation (39). Moreover, broader social determinants—such as perceived social support, access to community resources, and societal attitudes toward aging—may further shape suicidal ideation among older adults (30, 39, 42, 43). These patterns indicate a broader transnational need for culturally sensitive mental health and suicide prevention interventions.

Consistent with earlier studies (5, 18, 30), depression emerged as the strongest predictor of suicidal ideation in the present study. Depression in late life is often influenced by multiple risk factors, including social isolation, physical illness, and experiences of loss (17). As depressive symptoms intensify, older adults may perceive suicide as the only escape from suffering or as a way to avoid being a burden, especially in cultural contexts that value independence and self-sufficiency (18). These findings reinforce the importance of early screening and intervention for depression in late life.

With neural aspect, some studies have identified functional abnormalities in brain regions implicated in emotion regulation and cognitive control among depressed older adults. Altered activity in the dorsolateral and ventrolateral prefrontal cortex has been observed in individuals experiencing suicidal ideation, potentially reflecting impaired executive function and emotional regulation (11, 12). Additionally, increased connectivity within the default mode network has been associated with self-referential rumination, a cognitive style linked to depressive symptoms (12). In sum, depression in older adults cannot be adequately understood through a purely psychological lens. Rather, it requires a multidimensional framework that incorporates somatic health, functional capacity, and underlying neural mechanisms. The integration of psychological, physiological, and neurobiological perspectives might provides a more comprehensive understanding of depression and suicide risk in aging populations, and highlights the need for interdisciplinary approaches to prevention and intervention.

This study also confirmed protective roles of self-esteem and life satisfaction in reducing suicidal ideation. Prior research has suggested that internal psychological resources, such as a positive self-concept and a sense of life meaning, play more influential roles than external factors in preventing suicide among older adults (20, 21, 25). Higher levels of self-esteem allow individuals to better regulate emotional responses to stress and maintain resilience. Similarly, life satisfaction is closely associated with a sense of purpose and contentment, both of which can serve as buffers against hopelessness. Older adults may even report higher baseline levels of life satisfaction due to their ability to emotionally adapt and reframe challenges (4), suggesting the potential for targeted interventions to leverage these psychological strengths.

These findings highlight the need for multidimensional intervention strategies that not only target clinical symptoms, but also address broader social and psychological factors. Based on these results, a comprehensive policy approach that includes routine community-based mental health screening, development of tailored psychological support programs, expansion of intergenerational and community engagement initiatives, and public campaigns to reduce stigma around mental health in later life is recommended. Furthermore, policies supporting end-of-life planning and dignified aging may help alleviate fears of dependency, a factor closely linked to suicidal ideation. By implementing these strategies, society can move toward a more proactive, compassionate, and culturally sensitive framework for suicide prevention among the older adults.

Despite its contributions, this study has several limitations. First, as a cross-sectional analysis, it could not establish causal relationships between variables. Second, data were collected via self-report questionnaires, which might be subject to social desirability bias or inaccuracies in self-assessment. Finally, while this study used large-scale panel data, suicidal ideation remains a complex and multifaceted construct that may not be fully captured by survey-based measures. Future longitudinal studies are needed to better understand causal pathways between depression, self-esteem, life satisfaction, and suicidal ideation. Additionally, qualitative approaches could help uncover cultural and existential dimensions of suicide risk that are difficult to measure quantitatively.

Nonetheless, findings of this study contribute to the growing body of literature on suicide prevention in later life and provide a valuable foundation for designing evidence-based community screening tools and culturally informed intervention strategies.

## Data Availability

Publicly available datasets were analyzed in this study. This data can be found at: https://www.koweps.re.kr.
